# Investigating bacteriophages as a novel multiple-hurdle measure against *Campylobacter*: field trials in commercial broiler plants

**DOI:** 10.1038/s41598-024-53365-w

**Published:** 2024-02-07

**Authors:** Katrin Bogun, Elisa Peh, Borris Meyer-Kühling, Jürgen Hartmann, Juliane Hirnet, Madeleine Plötz, Sophie Kittler

**Affiliations:** 1 Institute for Food Quality and Food Safety, University of Veterinary Medicine Hannover, Foundation, Hannover, Germany; 2 PHW-Zentrallabor, BWE-Brüterei Weser-Ems GmbH & Co. KG, Visbek, Germany; 3 MEGA Tierernährung GmbH & Co. KG, Visbek, Germany

**Keywords:** Bacteriophages, Applied microbiology

## Abstract

*Campylobacter* mitigation along the food production chain is considered effective for minimizing the public health burden of human campylobacteriosis. This study is the first combining different measures in a multiple-hurdle approach, using drinking water additives and feed additives in single and combined application schemes in commercial broiler plants. Broiler chickens in the study groups were naturally contaminated with *Campylobacter*. Application of an organic acid blend via drinking water, consisting of sodium propionate, potassium sorbate, and sodium diacetate, resulted in significant reductions of up to 4.9 log_10_ CFU/mL in fecal samples and in cecal samples at slaughter. The application of a phage mixture, consisting of *Fletchervirus* phage NCTC 12673 and *Firehammervirus* phage vB_CcM-LmqsCPL1/1, resulted in reductions of up to 1.1 log_10_ CFU/mL in fecal samples 1 day after dosing. The sole administration of curcumin via feed resulted in small and inconsistent reductions. In the group receiving a combination of all tested measures, reductions of up to 1.1 log_10_ CFU/mL were observed. Based on the results of our field trials, it was shown that both the sole application and the combined application of mitigation measures in primary production can reduce the *Campylobacter* load in broiler chickens, while no synergism could be observed.

## Introduction

In 2021, campylobacteriosis was the most commonly reported infection with 127,840 confirmed cases^1^. *Campylobacter (C.) jejuni* and *C. coli* are the most important species^2^. During processing of chicken carcasses at slaughter, cross-contamination may occur from intestinal content and the bird’s skin or feathers^3,4^. It is estimated that a 3-log_10_ reduction in *Campylobacter* concentration in the ceca could reduce the risk of human campylobacteriosis arising from broiler meat consumption by 58%^5^.

Several mitigation measures have been investigated in the past targeting the poultry meat production line, comprising different stages of the food chain^3,6–8^. Applying mitigation measures during primary poultry production can have a major impact on the entry of *Campylobacter* during slaughter and processing^9^.

Organic acids, plant extracts, and bacteriophages are mitigation measures under discussion for *Campylobacter* reduction^7^. Organic acids are naturally derived organic compounds with acidic properties and approved for use in animal production^10–13^. Previous in vitro studies show that a combination of different organic acids has advantages over single administration, for example, the concentrations of the individual organic acids can be reduced in some combinations in which synergistic activities occur^14–16^. However, only a few investigations have addressed the application of combined organic acids^11,17,18^. In the present study, we build on a systematically developed and tested organic acid blend^11,14^.

Curcumin is a bioactive polyphenol, which is extracted from rhizomes of *Curcuma longa*^19^. Various studies demonstrated the pharmacologic properties of curcumin, such as antioxidant, anti-inflammatory, and antimicrobial effects^20–23^. However, to our knowledge, this is the first in vivo study investigating the antimicrobial activity of curcumin under commercial conditions^24–26^.

Bacteriophages (phages) are viruses that specifically infect certain bacterial cells^27^. Application of *Campylobacter*-specific phages via drinking water showed promising reductions of more than 3.2 log_10_ CFU/mL in commercial settings^28^. In a study by Chinivasagam et al.^29^, the use of phages in Australian broilers achieved reductions of up to 3 log_10_ CFU/g in one barn. Phages belonging to group II, predominantly classified as *Firehammervirus*, recognize their hosts by targeting the flagellum and are capable of infecting both *C. coli* and *C. jejuni*. Group III phages, classified as *Fletchervirus*, attach to the host's capsular polysaccharides and exclusively infect *C. jejuni*^30–32^. According to previous studies, a combination of both phage groups was most effective for *Campylobacter* mitigation^33^. Hammerl et al.^33^ observed higher *Campylobacter* reductions after applying mixtures of phages from both groups compared to application of single phages. However, there are few data on the application of a phage mixture under commercial conditions and this study is the first combining different measures in a multiple-hurdle approach^34–36^.

Various studies have investigated mitigation measures at different stages along the food chain in the past but none of the measures seem suitable for effectively reducing *Campylobacter* in a single application^37^. Measures can be preventive to prevent introduction of pathogens (such as biosecurity measures or preventive application of certain feed additives), or interventive to reduce the load in flocks that have been colonized already. A promising approach is the combined application of different measures to achieve an accumulation of reducing effects in a multiple-hurdle approach^38^. To our knowledge, this is the first study testing combined intervention measures in primary production under commercial conditions. For this purpose, organic acids, curcumin, and a phage mixture were applied in commercial broiler fattening plants.

## Results

Two field trials were carried out in commercial broiler fattening plants, each involving control and experimental groups with similar conditions. The field trials aimed to test treatments including phages, organic acids, and curcumin on chickens that were naturally colonized with *Campylobacter*.

### Field trial design 1: Multiple-hurdle application of phages, organic acids, and curcumin significantly reduced *Campylobacter* load in commercial broiler flocks

**Figure 1:**
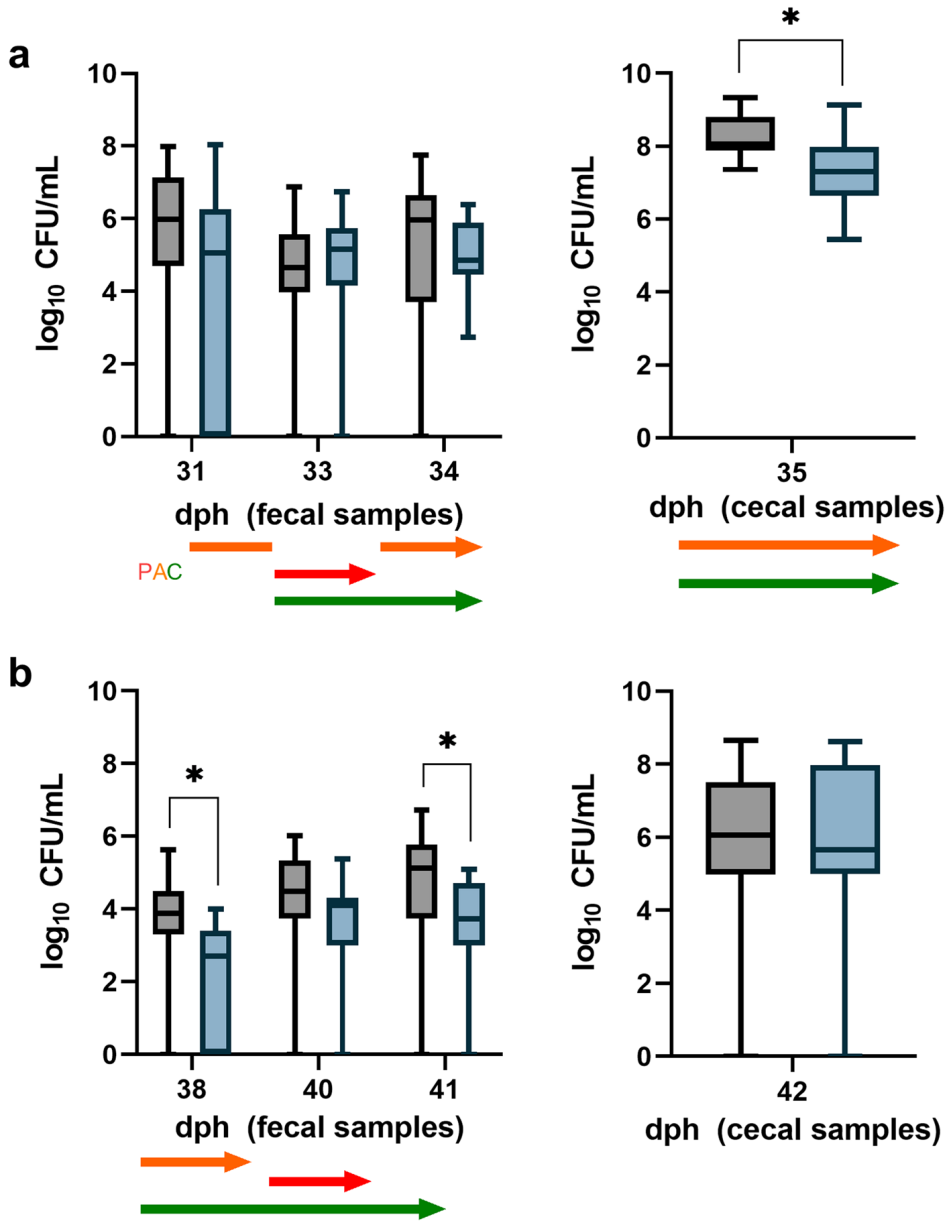
Comparison of *Campylobacter* counts of a control group (black boxplots) and experimental group PAC (turquoise boxplots) in field trial design 1. Birds in the experimental group PAC received a phage mixture (1.0 × 10^7^ PFU/bird), an organic acid blend via drinking water, and curcumin (160 mg/kg) via feed. Treatment of experimental group PAC: orange arrow = organic acid blend, red arrow = phage mixture, green arrow = curcumin. (**a**) *Campylobacter* counts in fecal and cecal samples until thinning. At day 31, samples were taken first and afterwards dosing was started. (**b**) *Campylobacter* counts of fecal and cecal samples after thinning until slaughter (n = 19). Boxplots indicate minimum, maximum, upper and lower quartile, and median values (n = 19). Significance levels (P values) determined by Mann–Whitney U test are indicated with * (P < 0.05). dph = days post hatch.

The absence of phages was confirmed at day 26 post hatch (dph) by overlay plaque assay when *Campylobacter* was first detected in the flocks. At 33 dph, dosage of curcumin via feed and first phage application were initiated. Organic acids were applied via drinking water starting at 31 dph, but paused at 33 dph for phage application. The calculated phage concentration at day 33 post hatch was 2.16 × 10^7^ plaque forming units (PFU)/bird and before final slaughter at 40 dph 1.01 × 10^7^ PFU/bird. The group receiving a combination of phages, organic acids, and curcumin will be abbreviated as “PAC” in the following. *Campylobacter* load of fecal and cecal samples during and after multiple-hurdle application are displayed in Fig. 1.

At first sampling before treatment (31 dph), control group samples harbored 0.9 log_10_ CFU/mL higher *Campylobacter* concentrations than the experimental PAC group samples (5.1 log_10_ CFU/mL) and only a slight difference of 0.5 log_10_ CFU/mL was observed between the groups at day 33 post hatch. Interestingly, 1 day after first phage application (34 dph), the median *Campylobacter* count in the PAC group was 1.1 log_10_ CFU/mL lower compared to the control. A significant reduction of 0.8 log_10_ CFU/mL in *Campylobacter* counts was also observed in cecal samples from subsequent thinning (35 dph). In fecal samples at 38 dph, a significant reduction in *Campylobacter* concentration of 1.2 log was observed in the experimental PAC group. The term “thinning” describes a partial depopulation one week before final slaughter in order to give space for the final stage of growth. At day 40 post hatch, *Campylobacter* counts were by 0.4 log_10_ CFU/mL lower in the PAC group, but this difference was not significant. After 41 dph, subsequent to the second phage application at final slaughter, *Campylobacter* counts in fecal samples were reduced significantly by 1.4 log_10_ CFU/mL. Similar results of 0.4 log_10_ CFU/mL reduction were observed in cecal samples from final slaughter.

In a two-factor analysis of variance, group and sampling day had a significant effect on the *Campylobacter* concentration, but there was no interaction between the variables. This is consistent with the graphical results, which showed similar curve shapes, but lower *Campylobacter* colonization levels associated with the applied measures (groups) and sampling day.

### Field trial design 1: Application of organic acids alone significantly reduced *Campylobacter* load by up to 4.9 log in fecal samples (33 dph)

**Figure 2:**
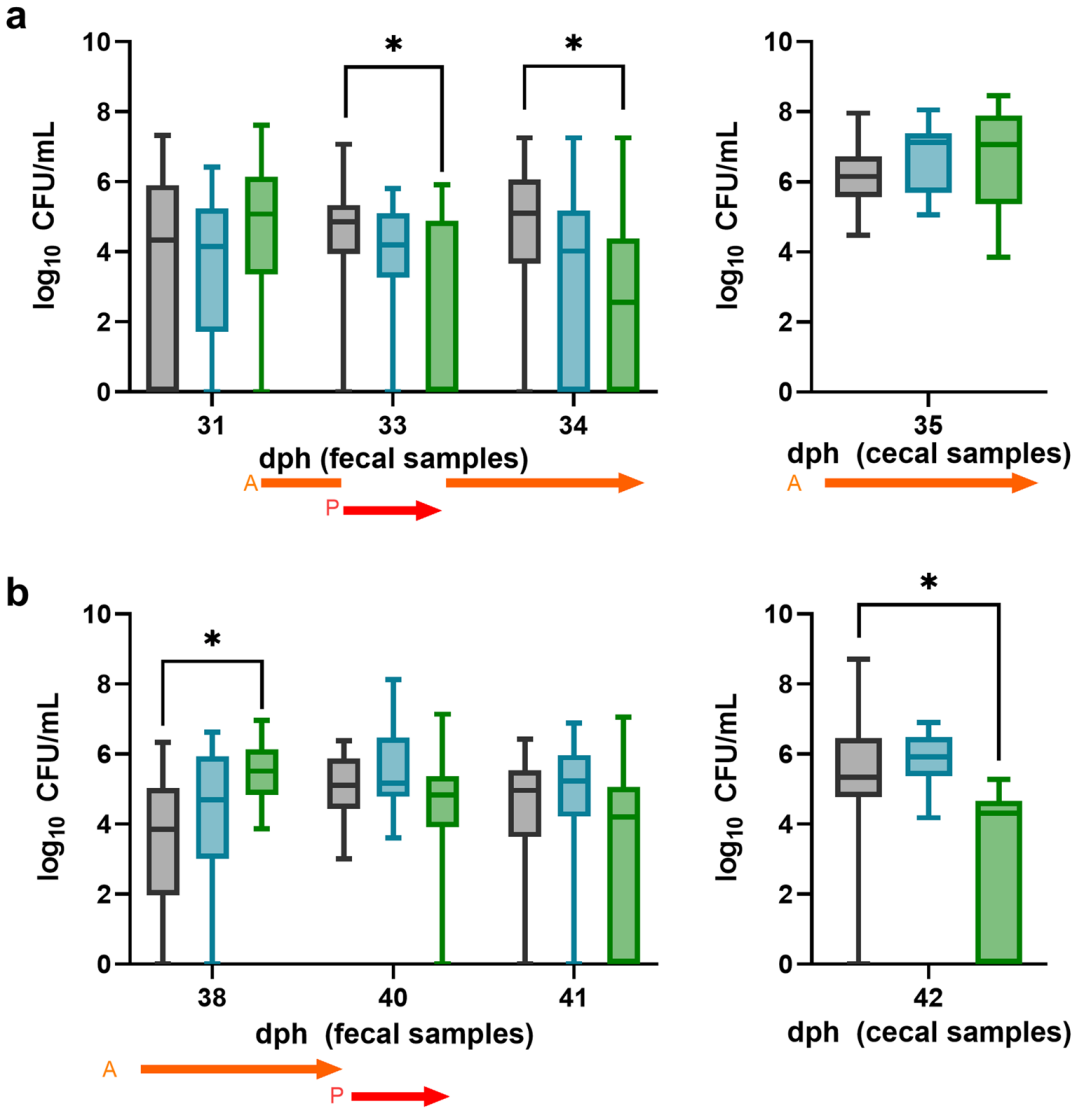
Comparison of *Campylobacter* counts of a control group (black boxplots) and group A (green boxplots), receiving organic acids via drinking water and group P (turquoise boxplots), receiving a phage mixture (8.94 × 10^6^ PFU/bird) via drinking water. Treatment of experimental groups: orange arrow = organic acid blend in group A, red arrow = phage mixture in group P. (**a**) *Campylobacter* counts in fecal and cecal samples until thinning. At day 31, samples were taken first and afterwards dosing was started. (**b**) *Campylobacter* counts of fecal and cecal samples after thinning until slaughter (n = 19). Boxplots indicate minimum, maximum, upper and lower quartile, and median values (n = 19). Significance levels (P values) determined by Kruskal–Wallis test are indicated with * (P < 0.05). dph = days post hatch.

The group that received an organic acid blend will be abbreviated as “A” in the following. In group A and its control, *Campylobacter* was detected 30 dph, and subsequently an organic acid blend was applied via drinking water. *Campylobacter* loads in fecal and cecal samples are depicted in Fig. 2 (green boxplots). Significant reductions of 4.9 log_10_ CFU/mL in fecal samples occurred 2 days after dosing started. At 34 dph, a significant difference of 2.55 log_10_ CFU/mL was still detected compared to the control. No *Campylobacter* reduction was detected in fecal and cecal samples from thinning until final slaughter. Subsequent to thinning, a linear decrease in *Campylobacter* colonization was observed in the experimental group A. However, due to the high initial levels this resulted in differences to the control group of a maximum of 0.3 (40 dph) and 0.8 (41 dph) log_10_ CFU/mL. At final slaughter, a significant reduction of *Campylobacter* of 1.0 log_10_ CFU/mL was detected in cecal samples of the experimental birds compared to the control birds.

### Field trial design 1: Application of phages alone reduced ***Campylobacter*** load by up to 1.1 log_10_ CFU/mL

A phage mixture comprising two phages was applied 2 days before thinning and before final slaughter in the experimental group, which will be abbreviated as “P” in the following. *Campylobacter* concentrations in this experiment are shown in Fig. 2 (turquoise boxplots).

Despite the fact that before phage application the median of the control group was 0.65 log_10_ CFU/mL higher compared to the experimental group P, application of a phage mixture via drinking water resulted in reductions of up to 1.1 log_10_ CFU/mL in fecal samples 1 day after application compared to the control. Nonetheless, no reduction was observed in cecal samples taken at the thinning process, where the median of the experimental group P was 0.9 log_10_ CFU/mL higher compared to the control group. Likewise, a higher median in group P was observed after thinning (38 dph). At day 40 post hatch, *Campylobacter* counts increased in both groups, resulting in comparable colonization levels (0.1 log_10_ CFU/mL difference). At day 41 post hatch, 1 day after phage application before final slaughter, a decrease of 0.5 log_10_ CFU/mL compared to the previous day was observed in the experimental group P (Fig. 2). Compared to the control group, the median in the experimental group P at day 41 post hatch was still 0.3 log_10_ CFU/mL higher.

### Field trial design 2: Curcumin did not lead to significant and consistent reductions of *Campylobacter* in commercial broiler flocks

The literature search for selecting the right curcumin dose to be used focused on studies in which curcumin was used as a feed additive for poultry. From a total of 24 selected studies, 14 focused on curcumin in the context of poultry and three of them investigated the antibacterial potential of curcumin as a feed additive for broilers and laying hens. In a study by da Rosa et al.^24^, curcumin was used as a feed additive for laying hens (200 mg/kg) and resulted in lower total bacterial counts, *E. coli* counts, and total coliform counts in both feces and eggs even at day 42.

**Figure 3:**
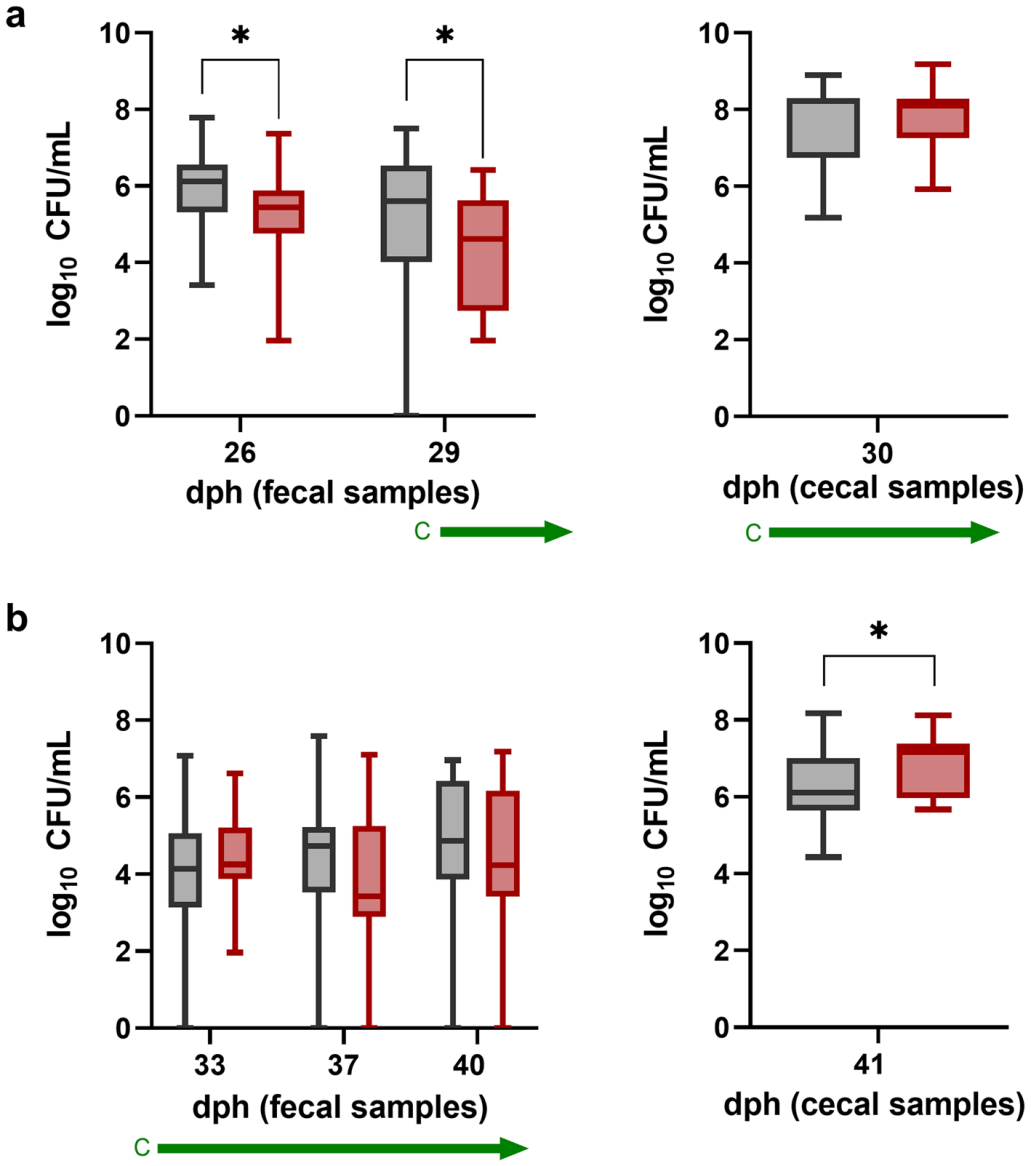
Comparison of *Campylobacter* counts of a control group (black boxplots) and group C (red boxplots) receiving curcumin (160 mg/kg) via feed. Treatment of experimental group: green arrow = curcumin. (**a**) *Campylobacter* counts in fecal and cecal samples until thinning. (**b**) *Campylobacter* counts of fecal and cecal samples after thinning until slaughter (n = 19). Boxplots indicate minimum, maximum, upper and lower quartile, and median values (n = 19). Significance levels (P values) determined by Mann–Whitney U test are indicated with * (P < 0.05). dph = days post hatch.

In our study, starting at day 29 post hatch, curcumin was applied daily via feed at a concentration of 160 mg/kg. The experimental group that received curcumin will be abbreviated as “C” in the following. Results are shown in Fig. 3.

Application of curcumin via feed in the experimental group C did not result in a significant and consistent reduction of *Campylobacter* counts. In fecal samples 3 days prior to administering curcumin, *Campylobacter* counts were significantly lower in group C compared to the control group. At day 29 post hatch, when curcumin dosing started, *Campylobacter* counts were still significantly lower. However, in cecal samples taken at the thinning slaughter, the median of *Campylobacter* load in the experimental group C was only a non-significant 0.1 log_10_ CFU/mL lower than the control. In cecal samples after slaughter, *Campylobacter* counts in the experimental group C were even significantly higher compared to the control. Using a two-factor analysis of variance, no interactions were detected for the sampling day and group, suggesting a similar development of *Campylobacter* load in both groups.

### Switch of *Campylobacter* strains was detected by multi-locus sequence typing (MLST) analysis in the phage group

Representative isolates from samples of all groups from field trials 1 and 2 were identified as *Campylobacter jejuni* using an MALDI-Biotyper^®^. No increasing impact of thinning on the amounts of *Campylobacter* colonization was detected in any of the flocks examined in the two field trials.

To determine whether different *Campylobacter* strains occurred during the field trials, multi-locus sequence typing analysis (MLST) was performed with 14 representative isolates.

In the first field trial, two sequence types (ST) were detected using MLST. In experimental PAC group and the respective control group, ST-353 was detected at the beginning and end of the field trial. In contrast, ST-1034 was present in control group A/P, while in the respective experimental group A, ST-353 was present. In the experimental group P belonging to control group A/P, ST-1034 was detected in all samples of experimental group P up until 1 day after the first phage application. After thinning (38 dph), only ST-353 was detected in the experimental group P.

In the second field trial, only ST-353 was detected in both groups at the beginning and end of the trial.

### Phages were present in samples after phage application and during slaughter

In all groups, collected fecal and cecal samples were further examined for the presence of phages. As hosts for phage detection, two *Campylobacter* strains *C. jejuni* strain NCTC 12662 and *C. coli* strain NCTC 12667 were used.

In field trial design 2, in which curcumin was applied, the absence of phages was confirmed in fecal as well as cecal samples at all sampling times. In field trial design 1, the absence of phages was shown in all groups before the first phage application. Directly after both phage applications, phages were detected in drinking water in the experimental group P. However, no phages were detected in fecal or cecal samples in the experimental group P thereafter. In the experimental group PAC, phages were detected in drinking water after both phage applications. Nevertheless, phage detection in fecal samples occurred 1 day after phage application before final slaughter (41 dph) only. Phages were detected in two fecal samples with concentrations ranging from 2.3 log to 3.1 log PFU/mL. Thereafter, phages were detected in cecal samples at final slaughter (42 dph), at concentrations ranging between 2.0 log and 3.0 log PFU/mL.

In the respective control group, phages were detected in fecal samples at day 40 post hatch. Eight fecal samples harbored phage concentrations of 2.0 log to 5.9 log PFU/mL. One day later (41 dph), similar results were obtained, with phage concentrations ranging from 4.1 log to 6.4 log PFU/mL. In 12 cecal samples after final slaughter, phages were observed with counts ranging from 4.5 log to 5.9 log PFU/mL.

The phages detected in control group PAC were further examined in order to assess whether these phages were identical to the applied phages or other naturally occurring phages. For this purpose, restriction endonuclease analysis (REA) and pulsed-field gel electrophoresis (PFGE) were performed. The applied phages and the phages detected in control group PAC were examined. The applied phage NCTC 12673 is a *Fletchervirus* phage whose DNA is susceptible to cutting by HhaI. The phage vB_CcM-LmqsCPL1/1 (LmqsCPL1/1) is a *Firehammervirus* phage whose DNA is resistant to digestion with HhaI but can be digested by SwaI. The DNA of phages isolated from samples of control group PAC was sensitive to digestion by HhaI but resistant to SwaI. Thus, phages from control group PAC were identified as *Fletchervirus* phages. Based on the different restriction patterns and genome sizes (NCTC 12673: 21.8 kilobases (kb), phages of control group PAC: 7.1 kb, 84.1 kb, 84.9 kb), our results suggest that the phages occurring in control group PAC were not identical to the phages used in our phage mixture.

### Susceptibility to phages was slightly reduced only after the second 3-h application of phages for multiple-hurdle mitigation

To detect possible resistance development, representative *Campylobacter* isolates were examined for their susceptibility to the two phages used by means of Direct Spot Test assay combined with phage dilution series. Due to *Campylobacter*-negative samples, numbers of examined isolates varied.

At day 31 post hatch, the 14 tested isolates of experimental group P receiving the phages were 100% (14/14) susceptible to both phages used. Two days after phage application at day 33 post hatch, the susceptibility of isolates was slightly lower with 84% of the 19 tested isolates being susceptible to NCTC 12673 and 95% (18/19) to LmqsCPL1/1. However, after second phage application at day 40 post hatch, susceptibility in isolates sampled 42 dph increased again to 95% (18/19), being susceptible to NCTC 12673 and 100% (19/19) to LmqsCPL1/1.

In the experimental group PAC, 93% (14/15) of the isolates were susceptible to NCTC 12673 and 100% (15/15) to LmqsCPL1/1 at day 31 post hatch. At day 35 post hatch, 2 days after the first phage application, the susceptible proportion of isolates in regard to NCTC 12673 and LmqsCPL1/1 increased to 94% (15/16). Two days after phage application before final slaughter, only 56% (10/18) of the tested isolates were susceptible to NCTC 12673 and 83% (15/18) to LmqsCPL1/1.

## Discussion

Campylobacteriosis is the most commonly reported foodborne bacterial zoonosis, requiring effective mitigation measures throughout the food chain for public health^39^. Since single measures did not result in reliable reduction in practical settings, a multiple-hurdle approach for *Campylobacter* mitigation was investigated. To our knowledge, this is the first study testing this approach under field conditions in commercial broiler production in northern Germany.

In the multiple-hurdle experimental group PAC receiving organic acids, phages, and curcumin, reductions of up to 1.1 log_10_ CFU/mL were observed 1 day after first phage application. Improved reduction compared to the control was observed in cecal samples of group PAC compared to group P where no reductions in cecal samples were observed (see Fig. 2). Furthermore, stronger reduction compared to phages (group P) or curcumin (group C) was observed after combined treatment (see Fig. 1 day 35). The observed effect is supported by in vitro experiments, where the combination of our organic acid blend and phage mixture resulted in a stronger bacterial growth suppression compared to single application (see Supplementary Fig. S1). Since our study tested a multiple-hurdle approach for the first time, no literature is available testing similar combinations. However, using antibiotics and phages showed a synergistic effect^40,41^.

Surprisingly, the most significant reduction in *Campylobacter* load occurred after applying organic acids only, with reductions of up to 4.9 log_10_ CFU/mL in fecal samples after 2 days. Given the enormously large quantities of organic acid mixture, the formulation of the organic acid blend had to be adapted due to the poor solubility of benzoic and sorbic acids^11^. Precipitation of the acids was avoided by using the respective salts, sodium propionate, potassium sorbate, and sodium diacetate. However, previous studies showed that MIC values of organic acids were in a similar range compared to those of the respective salts^14,42,43^. In addition, similar bactericidal properties of organic acids and their respective salts were shown in vitro^44^, which justifies the use of salts for practical feasibility. Still, at day 41 post hatch drinking lines were blocked in group A due to precipitation, and dosing was discontinued until final slaughter at day 42 post hatch. While no reductions could be measured in subsequent cecal samples taken at the thinning slaughter, *Campylobacter* load in the cecal samples taken at final slaughter were reduced by 1.0 log_10_ CFU/mL. Direct comparison of fecal and cecal samples with respect to *Campylobacter* concentration is difficult because the bacterium resides primarily in cecal contents, where *Campylobacter* concentrations can reach approximately 10^9^ cells per gram^4,45,46^. Therefore, the two sample matrices were considered separately when evaluating this study. One possible reason for missing reductions in cecal samples after thinning could be the short application time. The organic acid blend used in this study was based on a systematic development and testing against *Campylobacter *in vitro and in vivo in a previous study that demonstrated the advantages of combining multiple acids over a single application^11,14^. Applying these acids in vivo resulted in consistent reductions in a previous trial^11^. Other studies suggest that mechanisms of antibacterial effects of organic acids occur by a modified microbiome rather than by direct effect on target bacterial cells^47^. However, no results on the effect of the blend on the chicken gut microbiome are available.

Another possible reason for the lack of reproducible *Campylobacter* reduction in cecal samples could be the decrease in the concentration of organic acids in the gastrointestinal tract of broiler chickens. This could be linked to studies in which promising in vitro results could not be achieved with the same substances used in vivo. A study by Hermans et al.^48^ showed the activity of organic acids against *Campylobacter *in vitro, but no reductions could be obtained in vivo. Despite a bactericidal effect of butyrate detected in vitro in a study by Van Deun et al.^42^, no reductions could be obtained after applying butyrate-coated micro-beads in vivo. However, since significant results occurred at final slaughter after organic acid application was stopped due to precipitation, concentration of organic acid does not seem to explain our results. A delayed reduction could have occurred due to changed gut microbiome after application.

While inconsistent reductions by organic acids were reported in previous studies, our study demonstrated that an application of organic acids in primary production can lead to significant *Campylobacter* reductions of more than 4 log_10_ CFU/mL. As a possible optimization of the poor solubility of benzoic and sorbic acid, raising the pH value to achieve a buffering effect could be investigated to further ensure solubility after diluting with drinking water. In addition, an interval application could be carried out, as is already partly practiced with deworming in the poultry sector. Alternating dosing and rinsing with drinking water could possibly prevent precipitation and subsequent blocking of drinking lines. Since blocking of drinking lines did not occur until 9 days after the first application of the organic acids, interval application or the application via feed are promising options.

Single application of a phage mixture via drinking water was investigated during the first field trial. An application 2 days before thinning and final slaughter resulted in reductions of up to 1.1 log_10_ CFU/mL in fecal samples 1 day after application but not in subsequent fecal and cecal samples. These results suggest that the second application did not provide any obvious advantages over a single-dose application.

Significant reductions in cecal samples of 1–3 log_10_ CFU/g were reported in field trials by Chinivasagam et al.^29^ after phage application. In three independent field trials by Kittler et al.^28^, varying results were observed. These results indicate that reductions achieved after phage application in field trials are difficult to reproduce and are influenced by many factors, thus promoting the multiple-hurdle approach for reliable *Campylobacter* mitigation.

A critical aspect for the efficacy of phage application is the presence of a susceptible host bacterium, as phages are very specific to a particular host^49^. In field trials of Chinivasagam et al.^29^, the detected *Campylobacter* strains were tested for susceptibility to 19 phages, and only stables with high susceptible strains were used. This procedure was not possible in our field trial due to late colonization of the flocks just before thinning and long preparation times for large amounts of phage mixture. However, even if late colonization of broiler flocks resembles practical field conditions in German broiler production, and pre-screening of susceptibility should be considered in future trials to achieve high efficacy of phages, in vitro susceptibility cannot always predict susceptibility under in vivo field conditions.

The limited effect of phages in vivo may suggest different physiological states of bacteria in vivo compared to in vitro or that external matrices hinder phage infection in vivo^50^.

In a study by Connerton et al.^51^, it was revealed that the reason for replacement of one genotype by another after phage application was the survival and growth of new genotypes. The MLST results suggest similar factors in our study, as there was a change in the predominant sequence type in group P after the first phage application. Phages should cover a broad host spectrum. Nonetheless, in field trials, one cannot predict which *Campylobacter* isolates will occur at the time of phage application and these might change during propagation in the flocks. Consequently, a combination of a *Firehammervirus* phage and a *Fletchervirus* phage was selected to cover a broad range of isolates. Susceptibility rates of isolates confirm the general advantage of this approach.

However, phage re-isolation was very low in both experimental groups with phage application. Similarly, in a field trial by Kittler et al.^28^, detection rates of phages were very low, with concentrations ranging between log_10_ 0.5 PFU/g to log_10_ 2.1 PFU/g compared to 2.0 PFU/mL to 3.1 PFU/mL in our study, while Chinivasagam et al.^29^ detected 5.6 log_10_ PFU/g during field trials.

At day 40 post hatch, phages were isolated from fecal samples of control group PAC and identified as native phages using PFGE. The entry of native phages into commercial broiler plants cannot be prevented by biosecurity measures because phages occur ubiquitously^27^. In a study by Chinivasagam et al.^29^, phages were also detected in a control group, replicating on a sensitive *Campylobacter* population during the last week before slaughter, not being present in the pre-screening. In a study by Kittler et al.^28^, phages were also detected in the control group, but in contrast to our study, these were regarded as contaminating phages from the experimental group. Although the detection of native phages in the control group complicated the evaluation of the results of our study, no reduction of *Campylobacter* load was observed during their presence, thus indicating a low impact on our study results (Fig. 1).

Our results demonstrate that parameters such as application time, duration of application, phage concentration, and large scale propagation of phages need to be further optimized to achieve significant reductions. A factorial design for optimized dosing concentrations of phages and organic acids could be considered in further trials.

The third component of our multiple-hurdle approach was curcumin. At a concentration of 200 mg/kg as a feed additive in laying hens naturally infected with *Escherichia coli* (*E. coli*), curcumin resulted in lower *E. coli* counts and total coliform counts^24^. Even with a lower dosage of 100 mg/kg in a study by Galli et al.^26^, lower bacterial counts were observed after 42 days. By using 50 mg/kg curcumin, significantly lower bacterial counts at day 21 were achieved, but these increased at day 44 in comparison to the control group^25^. Similarly, in our study, a decrease in *Campylobacter* counts was detected between 33 and 37 dph after applying curcumin, but not thereafter. While plant extracts are increasingly being studied due to the need for novel antimicrobial agents, curcumin as a feed additive has been studied mostly in the context of growth performance, which could be improved in poultry^52–55^. A possible explanation for the lacking *Campylobacter* reduction by curcumin could be its low concentration or low antibacterial activity against *Campylobacter*. Additionally, curcumin is considered to have low bioavailability and is rapidly excreted^56^.

Field trials can serve as an important tool for investigating mitigating measures under realistic conditions. A major difficulty is the comparison of experimental groups with control groups after natural *Campylobacter* infection. In our field trials, all groups were located on the same farm and conditions for all groups were as identical as possible. Experimental and control groups were matched based on the date of initial *Campylobacter* detection. Thus, the conditions in field trials were kept as practical as possible, but also as comparable as possible. No changes in body weight gain, feed, and water uptake were noticed by the farmers.

Instead of a triplicate replication of combined PAC application, the effect of the substances was compared to the effect of single application in other groups. In replication trials, poor comparability due to different *Campylobacter* strains and study settings can be assumed as reported in previous studies^28^. Thus, comparable replicates of field trials in practice are not feasible due to differing bacterial isolates, properties, and colonization processes^18,28^. Our field trials based on a multiple-hurdle approach resulted in the findings that (1) using pre-harvest mitigation measures can reduce the *Campylobacter* load in primary production, (2) combined treatment resulted in higher reductions for certain substances compared to single application, and (3) a second phage application did not result in further advantages compared to single-dose application. Based on the knowledge obtained in our study, further research of combination treatments and timing of application is needed to maximize their effect and reproducibility and to further establish application in practice.

## Materials and methods

### Study design

Overall, two field trials were conducted at a commercial broiler fattening plant in Lower Saxony, Germany. Both field trials were carried out on the same farm, but in different barns. Information on flock size, breed, vaccination, and biosecurity measures are summarized in Supplementary Tables 1 and 2. In both field trials, identical conditions existed between control and experimental groups in terms of vaccinations, breed, and barn staff. Application of antibiotics differed between the groups (see Supplementary Tables 1 and 2), but none of the preparations used were agents that would affect *Campylobacter* colonization.

The first field trial included a total of five groups, including two control groups and three experimental groups. The animals in all groups were housed on the same day and were the same age. To ensure a natural colonization process of *Campylobacter*, only stables that were naturally colonized were selected. Since in the first field trial, two stables tested positive for *Campylobacter* at day 26 post hatch and three other stables tested positive at day 30 post hatch, two control groups were established for better comparison of the colonization progress (see Supplementary Table 1). Control group PAC, which tested positive for *Campylobacter* at day 26 post hatch was assigned to the experimental group that received a combination treatment of phages, organic acids, and curcumin (experimental group “PAC”). The three groups that tested positive at day 30 post hatch were divided into control group A/P and the two experimental groups receiving phages (“P”) and organic acids (“A”), respectively.

The second field trial included a control group and an experimental group (see Supplementary Table 2) receiving curcumin via feed (experimental group “C”). Both groups were located in two different buildings in order to provide separate feed and water supplies for both groups of animals. The animals in both groups were housed on the same day and were the same age. At day 25 post hatch, both groups were tested positive for *Campylobacter* by real-time PCR (Kylt, AniCon Labor GmbH, Höltinghausen, Germany) from fecal samples.

### Field trial design 1: experimental group organic acids (A)

In the first field trial, the application of an organic acid blend via drinking water and possible effects on *Campylobacter* colonization were investigated. Based on results of a previous study demonstrating synergistic effects against *Campylobacter* isolates, a mixture of sorbic acid, benzoic acid, propionic acid, and acetic acid was selected^14^. Due to the size of the experimental groups and the large quantities of organic acid blend required for them, an external company (KONIVET GmbH, Essen, Germany) was contracted to produce the organic acid blend, which was adapted because of the poor solubility of benzoic acid and sorbic acid. After excluding benzoic acid and using the respective salts of the three remaining acids, a mixture of sodium propionate, potassium sorbate, and sodium diacetate was prepared. The organic acid blend was dosed at 5 L per 1000 L of drinking water. The dosing was carried out daily from positive *Campylobacter* sampling (Supplementary Table 3) and was paused for 1 day during application of phages.

### Field trial design 1: experimental group phages (P)

For the application of phages in the field trials, an exemption was granted by the responsible authority (LAVES—Lower Saxony State Office for Consumer Protection and Food Safety, reference 41.3-63003-03/2022) in accordance with German law and ARRIVE guidelines (Animal Research: Reporting of In Vivo Experiments) were followed for reporting as far as they are applied for field trial conditions. The exemption permit in accordance with § 69 of the German Food and Feed Code (LFGB) in conjunction with Article 3 (2) of Regulation (EC) No. 1831/2003 covers the use of phages as feed additives.

The phage mixture consisted of a well-characterized *Fletchervirus* phage NCTC 12673 of the British phage typing scheme^57^ and a *Firehammervirus* phage LmqsCPL1/1^30^ that had been newly isolated.

For propagation of phage strains, *Campylobacter* was grown on sheep blood agar (Oxoid Deutschland GmbH, Wesel, Germany) and incubated at 41.5 ± 1 °C for 18 h under microaerobic conditions (5% O_2_, 10% CO_2_, and 85% N_2_). Subsequently, grown colonies were suspended in 10 mmol MgSO_4_. Density was adjusted to McFarland standard 3 (Densimat; bioMérieux). Phage NCTC 12673 was propagated on *C. jejuni* host strain NCTC 12661 and phage LmqsCPL1/1 was propagated on *C. coli* host strain Cc084610.

For propagation of phages, several flasks were each filled with 300 mL brain heart infusion broth (BHI), 18 mL inoculum (McFarland standard 3), and 6 mL of the respective phage suspension (log10 7.5 PFU/mL). Afterwards, flasks were swayed on an orbital shaker at 130 rpm under microaerobic conditions. After incubation, the contents of the flasks were centrifuged at 13,000×*g* for 10 min at 4 °C and filtered with VacuCap filtration devices (0.8/0.2 µm) (Pall Corporation, Port Washington, NY, USA) using a vacuum pump. Phage suspension was stored at 4 °C. Propagation of the two phages was performed separately and calculated volumes of both phages were mixed on the day of application.

The phage mixture was applied in experimental group P and PAC 2 days before thinning (33 days post hatch (dph)) and 2 days before slaughter (40 dph) (Supplementary Table 4). The drinking water supply was stopped 2 h before phage dosing to make the animals thirsty and increase phage uptake. Dosing of phages into the drinking lines was carried out over a 3-h period for each application.

### Field trial design 1: experimental group combination of phages, organic acids, and curcumin (PAC)

After positive sampling for *Campylobacter*, animals in the experimental group PAC received curcumin via feed and organic acids and phages via drinking water (Supplementary Table 3). The acid application was paused on the 2 days of phage application (33 dph and 40 dph).

### Field trial design 2: experimental group curcumin (C)

In both field trials, the feed additive curcumin was applied as an extract (Turmeric Extract ME, Delacon Biotechnik GmbH, Engerwitzdorf, Austria) containing 20–30% curcuminoids. When determining the concentration to be used, both the legal framework and an intensive literature research were considered with the goal of reducing *Campylobacter* colonization in the intestinal tract of broilers. The literature search focused on publications in which curcumin had been used as a feed additive for poultry. Subsequently, the research was continued in more detail and the respective concentration of curcumin was examined in the context of the following aspects: (1) possible bacterial reductions, (2) sensory examinations of poultry meat after feeding curcumin, (3) effects on growth performance.

Based on the current study situation and legal framework in Germany, a concentration of 160 mg/kg feed was targeted. For this purpose, 800 mg/kg of the above-mentioned turmeric extract, which contained approximately 20% curcuminoids, was dosed. In the second field trial, the reduction potential against *Campylobacter* was investigated when curcumin was administered alone (Supplementary Table 4). In the first field trial, curcumin was applied as a feed additive in combination with a phage mixture and an organic acid blend administered via drinking water.

### Sample collection

An overview of sample collection is shown in Supplementary Table 3 (field trial 1) and Supplementary Table 4 (field trial 2). A total of 19 fresh fecal samples per group were collected with sterile gloves from different locations in the stable and sealed in sterile plastic bags. In addition, 19 ceca per group were collected from the slaughter chain from commercial poultry slaughterhouses after evisceration. Moreover, water samples were collected from the beginning, middle, and end of the drinking lines. All samples were transported to the laboratory under chilled conditions at approximately 4 ± 2 °C and analyzed within a maximum of 24 h.

### Microbial analysis

Quantitative analysis of *Campylobacter* was performed in accordance with DIN EN ISO 10272-2:2017. For examination of fecal samples, 1 g of each sample was added to 9 mL of sodium chloride peptone buffer (NaCl, 8.5 g/L; peptone, 1 g/L). From this, log_10_ serial dilutions up to 10^–8^ were prepared in sodium chloride peptone buffer. Subsequently, 0.1 mL of each dilution was plated on CCDA plates (Oxoid Deutschland GmbH) in duplicate and incubated at 41.5 ± 1 °C for 48 h under microaerobic conditions (5% O_2_, 10% CO_2_, and 85% N_2_). Confirmation of presumed colonies was done by means of positive oxidase and catalase testing and evaluation of cell morphology and motility via microscopy. Of each positive sample, two isolates were collected and frozen in cryotubes (cryobank vials; skimmed milk) for subsequent examinations.

Cecal samples were collected by aseptically opening intestines from the slaughterhouse and weighing 1 g of cecal content per sample into 9 mL of sodium chloride peptone buffer. Analysis and confirmation were performed as described above.

### Further examination of representative isolates

For species identification, representative isolates from both field trials were analyzed by matrix-assisted laser desorption ionization-time of flight mass spectrometry (MALDI-TOF MS) (Microflex LT/SH MALDI-TOF, Bruker Daltonics GmbH & Co. KG, Bremen, Germany).

Furthermore, two representative isolates from each group (one isolate at the beginning of the field trial and one isolate directly before slaughter) were characterized with multi-locus sequence type (MLST) analysis. MLST was performed as described elsewhere^58,59^. Amplification was performed for all seven loci: aspA (aspartase), glnA (glutamine synthetase), gltA (citrate synthase), glyA (serine hydroxyl methyltransferase), pgm (phosphor glucomutase), tkt (transketolase), and uncA (ATP synthase alpha subunit), followed by purification (QIAquick PCR & Gel Cleanup Kit, Qiagen GmbH, Hilden, Germany). Sequencing reactions were carried out by Eurofins MWG Operon GmbH (Ebersberg, Germany). The Pub MLST database was used for analysis after sequences had been aligned with Clustal Omega^60^.

To determine susceptibility properties of isolates from the experiment, representative isolates from fecal and cecal samples were obtained on different sampling days. For this purpose, isolates from fecal and cecal samples of different time points were plated on sheep blood agar and analyzed by means of Direct Spot Test combined with a phage dilution series^30^. Susceptibility to phages was determined individually for phage NCTC 12673 and LmqsCPL1/1 and expressed as a percentage with absolute numbers in brackets.

### Enumeration and characterization of phages

For enumeration of phages, *C. jejuni* strain NCTC 12662 (for phage NCTC 12673) and *C. coli* strain NCTC 12667 (for phage LmqsCPL1/1) were used. One gram of cecal content or feces was added to 9 mL of SM buffer (5.8 g NaCl, 2.0 g MgSO_4_ × 7H_2_O, 50 mL 1 M Tris, adjusted to pH 7.5, filled up with distilled water to 1000 mL) and shaken overnight at 4 °C. Subsequently, centrifugation (13,000×*g* for 10 min at 4 °C) and filtration with 0.2 µm filters (Sarstedt AG & Co. KG, Nümbrecht, Germany) were performed. Drinking water samples were filtered only. After preparing a tenfold dilution series in SM buffer, phage enumeration was conducted using the double agar overlay method described by Fischer et al.^61^.

For calculating the number of phages applied per animal, the measured concentration in the drinking water and the volume of phage-dosed drinking water were used:

$$\text{PFU/bird=}\frac{\text{phage concentration in drinking water}\left[\frac{\text{PFU}}{{\text{mL}}}\right]\text{*volume of phage-dosed drinking water [mL]}}{\text{number of birds per flock}}$$

Phages were investigated by restriction endonuclease analysis (REA) using HhaI and SwaI and pulsed-field gel electrophoresis (PFGE) as described elsewhere^62^. However, for implementation of PFGE, GelRed (Biotium, Inc., Fremont, CA, USA) was used instead of ethidium bromide (EtBr).

### Statistical analysis

The required sample size was calculated using R software version 4.0.2 (R Core Team, 2020). Based on previous field trials^28^, a standard deviation of 1, delta = 1, α = 0.05, and β = 0.20 was assumed. To obtain homogeneity of variance, bacterial counts were transformed into log_10_ CFU/mL for microbial analysis^63^. The data of fecal and cecal samples were analyzed using the Mann–Whitney U test (field trial 2) and Kruskal–Wallis test (field trial 1) with GraphPad Prism 9.2.0 (GraphPad Software, San Diego, CA, USA). Pairwise comparisons were performed using Dunn's multiple comparison test. Furthermore, two-factor analysis of variance was performed with the main effects group and sampling day, and their interaction, using Tukey–Kramer adjustment as post hoc test with SAS Enterprise guide 7.1. (SAS Institute Inc., Cary, NC, USA). For all tests, p-values below 0.05 were considered statistically significant.

### Supplementary Information

Supplementary Information.

## Data Availability

Data will be made available on request.
