# Supplementary material for: Investigating bacteriophages as a novel multiple-hurdle measure against Campylobacter: field trials in commercial broiler plants
Source: Sci Rep. 2024 Feb 7;14:3182. doi: 10.1038/s41598-024-53365-w (PMC10850366; doi:10.1038/s41598-024-53365-w)
Supplement: Supplementary file 1 — Supplementary Information. [file 41598_2024_53365_MOESM1_ESM.pdf]

Supplementary Information

a

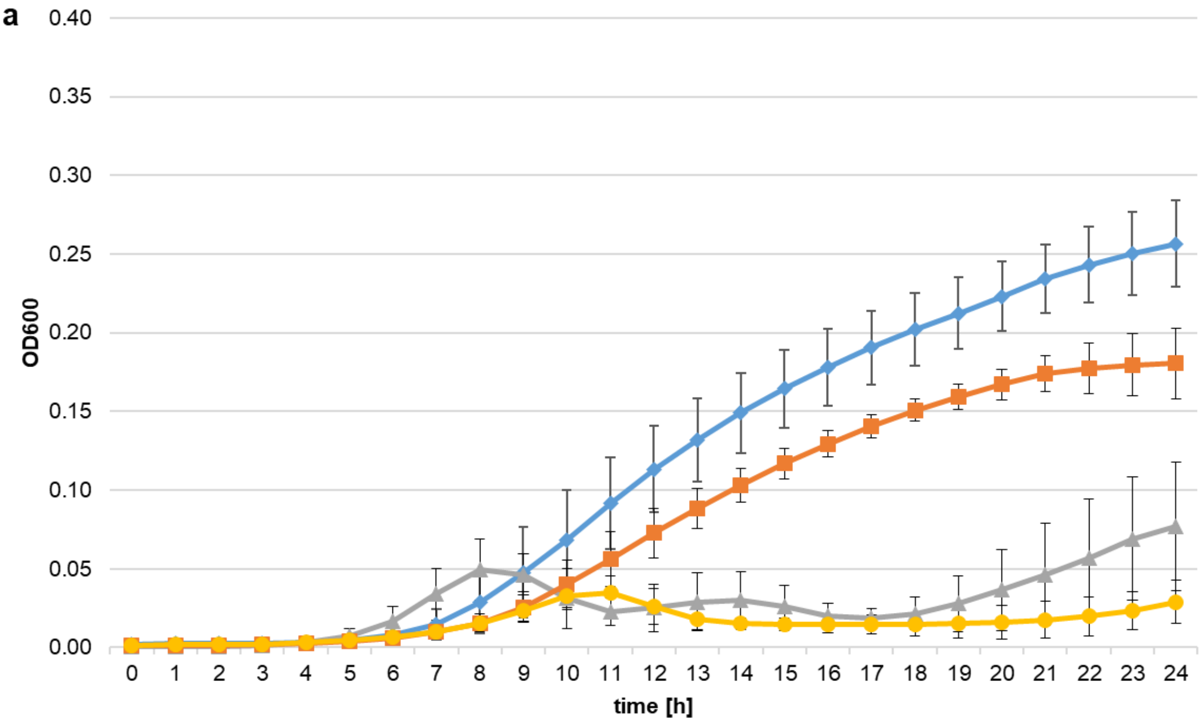

b

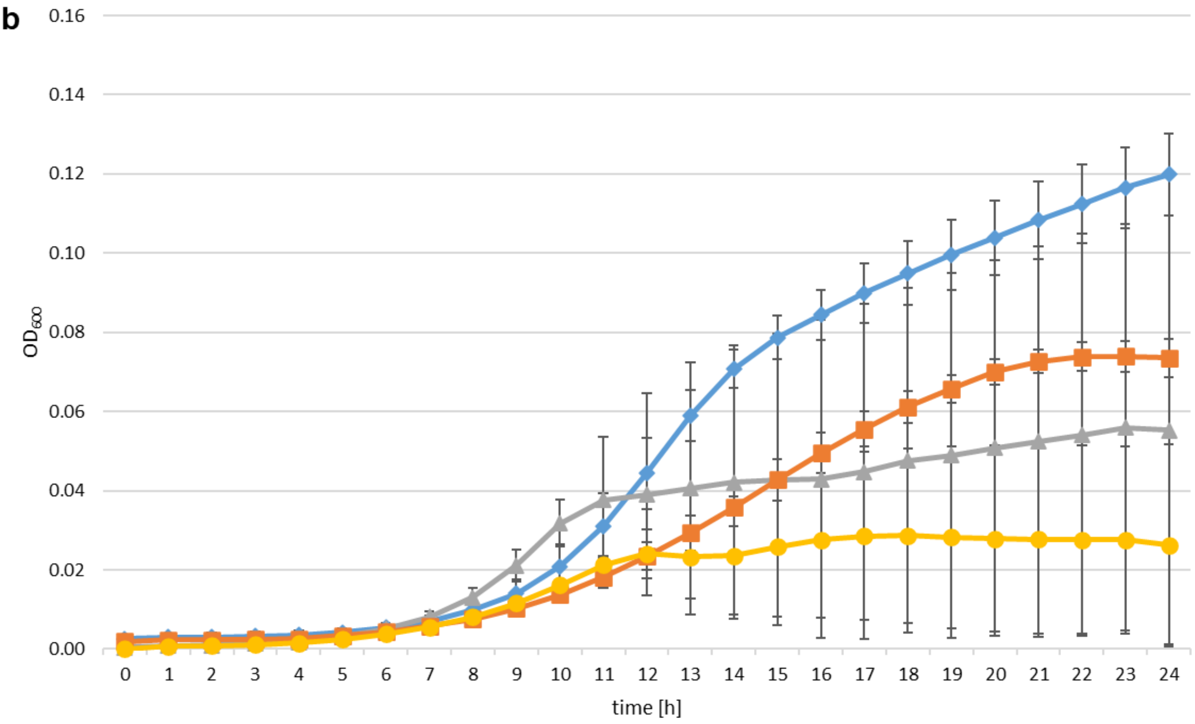

Supplementary Figure S1. Growth behavior of field isolates *C. coli* Cc7 (a) and *C. jejuni* Cj18 (b) in broth without added organic acids and phages ( ) and in broth supplemented with an organic acid blend ( ) (benzoic acid, sorbic acid, propionic acid and acetic acid) and a phage mixture ( ) consisting of NCTC 12673 and phage vB_CcM-LmqsCPL1/1 (Multiplicity of infection (MOI) of 0.001 for Cj18, MOI of 0.01 for Cc7), and a combination of organic acid blend and phage mixture ( ). Bacterial growth was monitored hourly by optical density (OD) absorbance measurements at 600 nm (OD_{600}) in a Tecan Spark® Plate Reader.

Supplementary Table 1. Study design of field trial design 1 with information on the stables, fattening period, breeding line, day of positive sampling, and biosecurity measures as well as vaccinations, feed additives, and medications.

**Field trial design 1**					
	**Control group PAC**	**Experimental group (PAC)[a]**	**Control group A/P**	**Experimental group (P)[b]**	**Experimental group (A)[c]**
Flock size<br>before thinning<br>before final slaughter	 26,000<br>19,646	 27,000<br>16,200	 30,500<br>18,792	 34,300<br>20,200	 30,800<br>23,807
Fattening period (days)	42	42	42	42	42
Breed	Ross 308				
Positive PCR result for *Campylobacter* spp.[d]	26 dph	26 dph	30 dph	30 dph	30 dph
Biosecurity<br>Building<br>Change of boots<br>Change of clothes	 separate<br>yes<br>no	 separate<br>yes<br>yes	 separate<br>yes<br>no	 separate<br>yes<br>yes	 separate<br>yes<br>no
Vaccination[e]	IBDV, NDV, IBV				
Feed additives	Minerals, Vit. A, D_3, E				
Early antibiotic therapy	Lincomycin, Spectinomycin 1 dph - 3 dph				
Antibiotic therapy (additional)	Colistin sulfate 35 dph - 37 dph		Penicillin 30 dph - 32 dph	Penicillin 27 dph - 29 dph	

[a]PAC = phages, organic acids, curcumin

[b]P = phages

[c]A = organic acids

[d]dph = days post hatch

[e]IBDV = infectious bursal disease virus, NDV = Newcastle disease virus, IBV = infectious bronchitis virus.

Supplementary Table 2. Study design of field trial design 2 with information on the stables, fattening period, breeding line, day of positive sampling, and biosecurity measures as well as vaccinations, feed additives, and medications.

Field trial design 2		
	**Control group**	**Experimental group (C)[a]**
Farm	1	1
Flock size (before thinning)	27,000	30,000
Fattening period (days)	41	41
Breed	Ross 308	
Positive PCR result for *Campylobacter* spp.[b]	25 dph	25 dph
Biosecurity		
Buildings of stables	different	different
Change of rubber boots	yes	yes
Change of clothes	no	no
Vaccination[c]	IBDV, NDV, IBV	
Feed additives	Minerals, Vit. A, D_3, E	
Early antibiotic therapy	Lincomycin, Spectinomycin 1 dph - 3 dph	

[a]C = curcumin

[b]dph = days post hatch

[c]IBDV = infectious bursal disease virus, NDV = newcastle disease virus, IBV = infectious bronchitis virus.

Supplementary Table 3. Timeline of application of organic acid blend and phage mixture in field trial design 1 and associated sampling.

Field trial design 1								
**Days post hatch**	**Control group PAC**	**Experimental group (PAC)[a]**			**Control group A/P**	**Experimental group (P)[b]**	**Experimental group (A)[c]**	**Sample collection[d]**
25								
26								
27								
28								
29								
30								
31				A			A	FS
32				A			A	
33		P	C			P		FS, WS
34			C	A			A	FS, WS
35			C	A			A	CS
36			C	A			A	
37			C	A			A	
38			C	A			A	FS
39			C	A			A	
40		P	C			P		FS, WS
41			C					FS, WS
42								CS

[a]PAC = phages, organic acids, curcumin

[b]P = phages

[c]A = organic acids

[d]FS = fecal samples, CS = cecal samples, WS = water samples.

Supplementary Table 4. Timeline of curcumin application in field trial design 2 and associated sampling.

Field trial design 2			
**Days post hatch**	**Control group**	**Experimental group (C)[a]**	**Sample collection[b]**
25			
26			FS
27			
28			
29		C	FS
30		C	CS
31		C	
32		C	
33		C	FS
34		C	
35		C	
36		C	
37		C	FS
38		C	
39		C	
40		C	FS
41			CS

[a]C = curcumin

[b]FS = fecal samples, cs = cecal samples.
